# An Asymptotic Analysis of the Malonyl-CoA Route to 3-Hydroxypropionic Acid in Genetically Engineered Microbes

**DOI:** 10.1007/s11538-020-00714-1

**Published:** 2020-03-06

**Authors:** Mohit P. Dalwadi, John R. King

**Affiliations:** 1 Mathematical Institute, University of Oxford, Oxford, OX2 6GG UK; 2 Synthetic Biology Research Centre, University of Nottingham, Nottingham, NG7 2RD UK; 3 School of Mathematical Sciences, University of Nottingham, Nottingham, NG7 2RD UK

**Keywords:** Multiscale, Bifurcation, 3HP, Kinetic model, Synthetic biology, Microbial production route, 34E10, 34E15, 37N25, 92C45

## Abstract

There has been recent interest in creating an efficient microbial production route for 3-hydroxypropionic acid, an important platform chemical. We develop and solve a mathematical model for the time-dependent metabolite concentrations in the malonyl-CoA pathway for 3-hydroxypropionic acid production in microbes, using a combination of numerical and asymptotic methods. This allows us to identify the most important targets for enzyme regulation therein under conditions of plentiful and sparse pyruvate, and to quantify their relative importance. In our model, we account for sinks of acetyl-CoA and malonyl-CoA to, for example, the citric acid cycle and fatty acid biosynthesis, respectively. Notably, in the plentiful pyruvate case we determine that there is a bifurcation in the asymptotic structure of the system, the crossing of which corresponds to a significant increase in 3-hydroxypropionic acid production. Moreover, we deduce that the most significant increases to 3-hydroxypropionic acid production can be obtained by up-regulating two specific enzymes in tandem, as the inherent nonlinearity of the system means that a solo up-regulation of either does not result in large increases in production. The types of issue arising here are prevalent in synthetic biology applications, and it is hoped that the system considered provides an instructive exemplar for broader applications.

## Introduction

3-Hydroxypropionic acid (3HP) is a platform chemical which can be converted into several valuable chemicals, for example acrylic acid and acrylamide (Werpy et al. 2004). There has been significant recent interest in introducing metabolic pathways to microorganisms in order to produce 3HP at industrially viable levels (Borodina et al. 2015; Chen et al. 2014, 2017; Kim et al. 2014; Son et al. 2017; Matsakas et al. 2018). The manipulation of metabolic pathways in microorganisms is an exciting avenue of research arising in synthetic biology. As enzyme production within an organism is genetically controlled, introducing specific genes allows an organism to produce chemicals that it could not previously. One of the most successful examples of a chemical synthesized by this procedure is amorpha-4,11-diene (a precursor to artemisinin, an antimalarial) from *Escherichia coli* (Martin et al. 2003). It can be expensive and time-consuming to introduce a new chemical pathway to a microorganism and optimize the production of a given metabolite. Moreover, adding and blocking pathways can have unintended consequences for the metabolism of the microorganism, and the experimental parameter space is extensive. Mathematical modelling allows systematic progress to be made in understanding a pathway and can significantly reduce the experimental parameter space that needs to be searched.

**Fig. 1:**
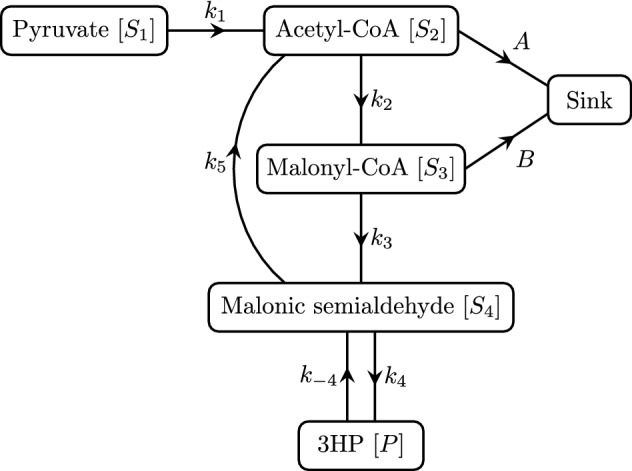
A schematic network diagram for the metabolic pathway we consider in this paper, from pyruvate to 3HP via malonyl-CoA. The arrows denote the direction of the reactions and are labelled by their respective catalytic constants (also referred to as turnover numbers). We only track the metabolites included in this figure and, specifically, not any involved in the acetyl-CoA or malonyl-CoA sinks. The subscript for a given enzyme $E_i$ (for $i = 1, \ldots , 5$) corresponds to the subscript of the related maximal reaction rate. The exception here is for the reversible reaction between malonic semialdehyde and 3HP, where both reactions use the enzyme $E_4$. Hence, $E_1$ corresponds to the pyruvate dehydrogenase complex (EC 1.2.4.1, EC 2.3.1.12, and EC 1.8.1.4), $E_2$ corresponds to acetyl-CoA carboxylase (EC 6.4.1.2), $E_3$ corresponds to malonyl-CoA reductase (EC 1.2.1.75), $E_4$ corresponds to 3-hydroxypropionate dehydrogenase (EC 1.1.1.298), and $E_5$ corresponds to malonate semialdehyde dehydrogenase (acetylating) (EC 1.2.1.18)

In Kumar et al. (2013), three thermodynamically feasible pathways from pyruvate to 3HP are suggested. In previous work, we investigated 3HP production via the $\beta$-alanine route using mathematical modelling (Dalwadi et al. 2017). Here, we are interested in mathematically modelling 3HP production via the malonyl coenzyme A (malonyl-CoA) route, with the aim of determining the most appropriate enzyme targets for regulation. We consider the reactions

1a $$\begin{aligned} {\text {Pyruvate}}&\xrightarrow {k_1} {\text {Acetyl-CoA}}, \end{aligned}$$

1b $$\begin{aligned} {\text {Acetyl-CoA}}&\xrightarrow {k_2} {\text {Malonyl-CoA}}, \end{aligned}$$

1c $$\begin{aligned} {\text {Malonyl-CoA} }&\xrightarrow {k_{3}} {\text {Malonic semialdehyde}} , \end{aligned}$$

1d $$\begin{aligned} {\text {Malonic semialdehyde}}&\mathop {\rightleftharpoons }\limits ^{k_4}_{k_{-4}} {\text {3HP}} , \end{aligned}$$

1e $$\begin{aligned} {\text {Malonic semialdehyde}}&\xrightarrow {k_{5}} {\text {Acetyl-CoA}}, \end{aligned}$$

1f $$\begin{aligned} {\text {Acetyl-CoA}}&\xrightarrow {A} {\text {Sink}}, \end{aligned}$$

1g $$\begin{aligned} {\text {Malonyl-CoA}}&\xrightarrow {B} {\text {Sink}}, \end{aligned}$$

where (1f) and (1g) represent the loss of acetyl-CoA and malonyl-CoA to other metabolic pathways, such as the citric acid cycle or fatty acid biosynthesis. We do not track any metabolites once they reach the sink. We show a schematic representation of this pathway in Fig. 1.

Our general aim is to determine how the system behaves as a function of its parameters, with our main goal being to understand how to maximize 3HP production while minimizing the levels of malonic semialdehyde, a toxic intermediate, where possible. To derive and solve our mathematical model, we make several modelling assumptions. We consider a system that is well mixed and thus spatially independent. This means that we are able to formulate a mathematical system in terms of ordinary differential equations in time, rather than partial differential equations in time and space. These differential equations require initial conditions, and we consider the case where pyruvate is instantaneously introduced to a system containing all of the relevant enzymes, but none of the intermediate metabolites. This assumption facilitates a mathematical analysis by reducing the number of unknown parameters in the system. Moreover, we follow the approach of Dalwadi et al. (2017) and Dalwadi et al. (2018) and investigate two simplified cases of pyruvate replenishment, which model the extremes of the actual time-dependent pyruvate replenishment. The first of these is continuous pyruvate replenishment, where the pyruvate is held at a constant concentration, which could represent a continuous culture, and the second is no pyruvate replenishment which could represent a batch culture. Understanding these extreme cases allows us to determine the key targets for enzyme regulation. Finally, we assume that the formation rate of enzyme complex production is much quicker than the rate of substrate consumption, and thus, the reaction rates are governed by Michaelis–Menten-type laws, the specific form of which we obtain from the literature.

To analyse the nonlinear governing equations that we derive, we use a combination of numerical and asymptotic methods. The latter enhances our physical insight into the underlying system and allows us to derive closed-form expressions for how the metabolite concentrations vary as functions of the experimental parameters. Asymptotic techniques (see, for example, Holmes 2012; Kevorkian and Cole 2013) allow us to largely bypass the issue of uncertainty in the parameters, as deriving analytic approximations of the dynamic metabolite concentrations only requires an understanding of the relative order of magnitude of each experimental parameter. Moreover, the nonlinear nature of the system means that a broad understanding of the system behaviour cannot be obtained by simply varying one parameter at a time and collecting system outputs, so asymptotic solutions allow for a quicker and more comprehensive understanding of the system. We note that there is a bifurcation in the asymptotic structure of the system for the case of continuous pyruvate replenishment, and we investigate this in terms of our goal of maximizing 3HP production.

In Sect. 2, we introduce a mathematical model to describe the reaction kinetics. We solve this system numerically and asymptotically, in Sect. 3 for the case with a continuous replenishment of pyruvate, and in Sect. 4 for the case with no replenishment of pyruvate. Finally, in Sect. 5 we discuss our results.

## Model Description

The method we use to set up our governing equations is similar to that of Dalwadi et al. (2018), but for a different metabolic network. Thus, to describe the dynamics of the network from pyruvate to 3HP, shown in Fig. 1, we obtain corresponding Michaelis–Menten-type reaction velocities from the literature. The resulting dimensional governing system is

2a $$\begin{aligned} \frac{\mathrm {d} [S_1]}{\mathrm {d} \tau }&= -\dfrac{k_1E_1K_1^i[S_1]}{K_1^i\left( [S_1]+ K_1^M\right) + [S_1][S_2]}, \end{aligned}$$

2b $$\begin{aligned} \frac{\mathrm {d} [S_2]}{\mathrm {d} \tau }&= \dfrac{k_1E_1K_1^i[S_1]}{K_1^i\left( [S_1]+ K_1^M\right) + [S_1][S_2]} - \dfrac{k_2E_2[S_2]}{[S_2]+ K_{2}^M} + \dfrac{k_{5}E_{5}[S_4]}{[S_4]+ K_{5}^M} - A[S_2], \end{aligned}$$

2c $$\begin{aligned} \frac{\mathrm {d} [S_3]}{\mathrm {d} \tau }&= \dfrac{k_2E_2[S_2]}{[S_2]+ K_{2}^M} - \dfrac{k_{3}E_{3}[S_3]}{[S_3]+ K_{3}^M} - B[S_3], \end{aligned}$$

2d $$\begin{aligned} \frac{\mathrm {d} [S_4]}{\mathrm {d} \tau }&= \dfrac{k_{3}E_{3}[S_3]}{[S_3]+ K_{3}^M} - \dfrac{k_4E_4[S_4]}{[S_4]+ K_4^M} + \dfrac{k_{-4}E_4[P]}{[P]+ K_{-4}^M} - \dfrac{k_{5}E_{5}[S_4]}{[S_4]+ K_{5}^M}, \end{aligned}$$

2e $$\begin{aligned} \frac{\mathrm {d} [P]}{\mathrm {d} \tau }&= \dfrac{k_4E_4[S_4]}{[S_4]+ K_4^M} - \dfrac{k_{-4}E_4[P]}{[P]+ K_{-4}^M}, \end{aligned}$$

where each variable is defined in Table 1. We will consider two extreme cases of system (2), corresponding to continuous and no replenishment of pyruvate, respectively, noting that the reality will lie somewhere between these two extremes. In (2), most of the reaction velocity terms are in standard Michaelis–Menten form, and we obtain the kinetic parameters for these reaction velocities from the corresponding references in Table 2. A lower-case *k* represents a catalytic constant, and an upper-case *K* represents a Michaelis (or Michaelis-type) constant. We include a modified Michaelis–Menten term for reaction (1a), which encompasses the uncompetitive inhibitory effect that acetyl-CoA has with pyruvate (Kresze and Ronft 1981a). Therefore, we use the standard form for uncompetitive inhibition for this reaction velocity (Yung-Chi and Prusoff 1973). Reactions (1f) and (1g) correspond to the aggregated loss of acetyl-CoA and malonyl-CoA, respectively, to other metabolic pathways present in the microorganism, such as the citric acid cycle or fatty acid biosynthesis. We model each of these aggregated losses as a sink with first-order reaction kinetics, using the parameters $A$ and $B$ to represent the strength of each sink for acetyl-CoA and malonyl-CoA, respectively. To get around the issue that these parameters are unknown, we will later consider a distinguished asymptotic limit (in a sense to be made formal later) where both of these sink reactions balance the other reactions. The parameters $E_i$, where $i =1, \ldots , 5$, denote the initial enzyme concentrations for the reactions they control, noting that the reversible reaction with maximal reaction rates $k_{4}$ and $k_{-4}$ are both controlled by $E_4$.

**Table 1:** Dimensional and dimensionless variable definitions

Original variable	Description	Dimensionless variable
$[S_1]$	Pyruvate	$[S_1]= S_0S_1$
$[S_2]$	Acetyl-CoA	$[S_2]= S_0S_2$
$[S_3]$	Malonyl-CoA	$[S_3]= S_0S_3$
$[S_4]$	Malonic semialdehyde	$[S_4]= S_0S_4$
$[P]$	3HP	$[P]= S_0P$
$\tau$	Time	$\tau = (S_0/k_1E_1) t$

**Table 2:** Parameter values

Dimensional	Organism	Range	Dimensionless
$k_1= 15 \, \mathrm {s}^{-1}$	*Lactococcus lactis* (Snoep et al. 1992)	4–$30 \, \mathrm {s}^{-1}$ (Kresze and Ronft 1981a, b; Snoep et al. 1992)	
$k_2= 12 \, \mathrm {s}^{-1}$	*Acinetobacter baumannii* (Alves et al. 2011)	12–$30 \, \mathrm {s}^{-1}$ (Alves et al. 2011; Hügler et al. 2003)	$\bar{k}_2= k_2E_2/ \epsilon k_1E_1= 11.4$
$k_{3}= 28 \, \mathrm {s}^{-1}$	*Sulfolobus tokodaii* (Alber et al. 2006)	3–$50 \, \mathrm {s}^{-1}$ (Hügler et al. 2002; Alber et al. 2006)	$\bar{k}_{3}= k_{3}E_{3}/k_1E_1= 0.19$
$k_4= 100 \, \mathrm {s}^{-1}$	*Nitrosopumilus maritimus* (Otte et al. 2015)	100–$120 \, \mathrm {s}^{-1}$ (Otte et al. 2015; Kockelkorn and Fuchs 2009)	$\bar{k}_4= k_4E_4/k_1E_1= 0.67$
$k_{-4}= 2.1 \, \mathrm {s}^{-1}$	*Nitrosopumilus maritimus* (Otte et al. 2015)	0.2–$2.1 \, \mathrm {s}^{-1}$ (Otte et al. 2015; Yao et al. 2010)	$\bar{k}_{-4}= k_{-4}E_4/\epsilon k_1E_1= 2$
$k_{5}= 20 \, \mathrm {s}^{-1}$	*Pseudomonas fluorescens* (Yamada and Jakoby 1960)	9–$20 \, \mathrm {s}^{-1}$ (Yamada and Jakoby 1960; Anderson and Magasanik 1971)	$\bar{k}_{5}= k_{5}E_{5}/k_1E_1= 0.13$
$K_1^M= 1 \, \mathrm {mM}$	*Lactococcus lactis* (Snoep et al. 1992)	0.13–$1 \, \mathrm {mM}$ (Kresze and Ronft 1981a; Pronk 1996; Snoep et al. 1992)	$\bar{K}_1^M= K_1^M/S_0= 0.5$
$K_1^i= 0.014 \, \mathrm {mM}$	*Saccharomyces cerevisiae* (Kresze and Ronft 1981a)	0.014–$0.018 \, \mathrm {mM}$ (Kresze and Ronft 1981a; Pronk 1996)	$\epsilon = K_1^i/S_0= 0.007$
$K_{2}^M= 0.01 \, \mathrm {mM}$	*Acinetobacter baumannii* (Alves et al. 2011)	0.01–$0.06 \, \mathrm {mM}$ (Alves et al. 2011; Hügler et al. 2003)	$\bar{K}_{2}^M= K_{2}^M/\epsilon S_0= 0.71$
$K_{3}^M= 0.04 \, \mathrm {mM}$	*Sulfolobus tokodaii* (Alber et al. 2006)	0.03–$0.1 \, \mathrm {mM}$ (Hügler et al. 2002; Alber et al. 2006)	$\bar{K}_{3}^M= K_{3}^M/\epsilon S_0= 2.86$
$K_4^M= 0.11 \, \mathrm {mM}$	*Nitrosopumilus maritimus* (Otte et al. 2015)	0.07–$0.11 \, \mathrm {mM}$ (Otte et al. 2015; Kockelkorn and Fuchs 2009)	$\bar{K}_4^M= K_4^M/\epsilon S_0= 7.86$
$K_{-4}^M= 7.9 \, \mathrm {mM}$	*Nitrosopumilus maritimus* (Otte et al. 2015)	7.9–$17 \, \mathrm {mM}$ (Otte et al. 2015; Yao et al. 2010)	$\bar{K}_{-4}^M= K_{-4}^M/ S_0= 3.95$
$K_{5}^M= 0.03 \, \mathrm {mM}$	*Pseudomonas fluorescens* (Yamada and Jakoby 1960)	0.03–$0.06 \, \mathrm {mM}$ (Yamada and Jakoby 1960; Anderson and Magasanik 1971)	$\bar{K}_{5}^M= K_{5}^M/\epsilon S_0= 2.14$
$A\quad [\mathrm {s}^{-1}]$			$\bar{A}= AS_0/ k_1E_1= \epsilon$
$B\quad [\mathrm {s}^{-1}]$			$\bar{B}= BS_0/ k_1E_1= 1/\epsilon$

To give an idea of the range of these parameter values, we provide data from several organisms. As we use an asymptotic analysis, it is the relative magnitude of these values, rather than their exact values, which are important. As discussed in the main text, we use the value $S_0= 2 \, \mathrm {mM}$, assume that $E_i/ E_1 = 0.1$ for $i \in \{2, \ldots 5 \}$, and we scale extreme parameter ratios with the small dimensionless parameter $\epsilon$, defined as the ratio of $K_1^i$ and $S_0$. As there is a distinguished asymptotic limit when $\bar{A}= O (\epsilon )$ and $\bar{B}= O (1/\epsilon )$, we use these scalings in our asymptotic analysis, choosing $\bar{A}= \epsilon$ and $\bar{B}= 1/\epsilon$ in our simulations

The initial levels of each metabolite present in the system are relatively unknown. Thus, we make a modelling choice to reduce the number of uncertain parameters in the system to facilitate a simplified analysis of the system. We use initial conditions corresponding to the case in which pyruvate is instantaneously introduced to a system containing all of the relevant enzymes, but none of the intermediate metabolites. That is, we use $[S_1](0) = S_0$, where $S_0$ represents the prescribed initial or typical level of pyruvate present in the system, with none of remaining metabolites initially present: $[S_2](0) = [S_3](0) = [S_4](0) = [P](0) = 0$.

We now nondimensionalize the system and exploit various small dimensionless parameter ratios to introduce a small parameter into the system, thus allowing us to write the system in terms of $$ O (1)$$ constants and one small parameter to quantify the relative size of each term. This is useful because kinetic parameters can vary between different environments, as shown in Table 2. To side-step the issues associated with this parameter uncertainty, we seek to quantitatively understand how the system behaves as these parameters vary by interrogating the system using an asymptotic analysis (see, for example, Holmes 2012; Kevorkian and Cole 2013).

We provide our nondimensional variable scalings in Table 1. Essentially, we scale each dimensional metabolite concentration with $$S_0$$, the initial concentration of pyruvate, and we scale time with $$S_0/(k_1E_1)$$, the characteristic time of the first reaction, which occurs between pyruvate and acetyl-CoA. To form the dimensionless parameters in the system, we first scale each rate constant with the rate constant of the first reaction, each Michaelis constant with the initial pyruvate concentration, and each enzyme concentration with the concentration of the first enzyme. Then, using the typical dimensional values in Table 2, we note that the parameter ratio $$\epsilon := K_1^i/S_0= 7 \times 10^{-3}$$ is very small. Hence, we scale the other extreme parameter ratios in our system with the small dimensionless parameter $$\epsilon $$, allowing us to write each additional dimensionless parameter as $$c \epsilon ^j$$, where *c* is an $$ O (1)$$ parameter (in practice, we take this to be between $$\epsilon ^{1/2}$$ and $$\epsilon ^{-1/2}$$), and *j* is an integer. This allows us to quantify the “smallness” of each parameter while allowing each parameter *c* to vary over approximately three orders of magnitude, yielding a system in terms of a small parameter. We take the typical concentration of pyruvate within a microorganism to be $$S_0= 2 \, \mathrm {mM}$$, and we assume that $$E_i/ E_1 = 0.1$$ for $$i \in \{2, \ldots 5 \}$$. We make this assumption since we expect the levels of pyruvate dehydrogenase complex (PDC), corresponding to $$E_1$$, to be more abundant than the other enzymes in this pathway since PDC is fundamental to cellular respiration in that it links glycolysis to the citric acid cycle. However, we emphasize that different values of $$E_i$$ can be considered if required by varying the appropriate dimensionless parameter. The resultant dimensionless parameters we use in our system are given in Table 2.

The dimensionless system is then given by

3a $$\begin{aligned} \frac{\mathrm {d} S_1}{\mathrm {d} t}&= -\dfrac{\epsilon S_1}{\epsilon \left( S_1+ \bar{K}_1^M\right) + S_1S_2}, \end{aligned}$$

3b $$\begin{aligned} \frac{\mathrm {d} S_2}{\mathrm {d} t}&= \dfrac{\epsilon S_1}{\epsilon \left( S_1+ \bar{K}_1^M\right) + S_1S_2} - \dfrac{\epsilon \bar{k}_2S_2}{S_2+ \epsilon \bar{K}_{2}^M} + \dfrac{\bar{k}_{5}S_4}{S_4+ \epsilon \bar{K}_{5}^M} - \bar{A}S_2, \end{aligned}$$

3c $$\begin{aligned} \frac{\mathrm {d} S_3}{\mathrm {d} t}&= \dfrac{\epsilon \bar{k}_2S_2}{S_2+ \epsilon \bar{K}_{2}^M} - \dfrac{\bar{k}_{3}S_3}{S_3+ \epsilon \bar{K}_{3}^M} - \bar{B}S_3, \end{aligned}$$

3d $$\begin{aligned} \frac{\mathrm {d} S_4}{\mathrm {d} t}&= \dfrac{\bar{k}_{3}S_3}{S_3+ \epsilon \bar{K}_{3}^M} - \dfrac{\bar{k}_4S_4}{S_4+ \epsilon \bar{K}_4^M} + \dfrac{\epsilon \bar{k}_{-4}P}{P+ \bar{K}_{-4}^M} - \dfrac{\bar{k}_{5}S_4}{S_4+ \epsilon \bar{K}_{5}^M}, \end{aligned}$$

3e $$\begin{aligned} \frac{\mathrm {d} P}{\mathrm {d} t}&= \dfrac{\bar{k}_4S_4}{S_4+ \epsilon \bar{K}_4^M} - \dfrac{\epsilon \bar{k}_{-4}P}{P+ \bar{K}_{-4}^M}, \end{aligned}$$

where $\epsilon$ is a small parameter. The dimensionless initial conditions are: $S_1(0) = 1$, $S_2(0) = 0$, $S_3(0) = 0$, $S_4(0) = 0$, and $P(0) = 0$.

We emphasize that the asymptotic sizes of $\bar{A}$ and $\bar{B}$ are not currently set, and that we have not yet scaled our dependent variables with $\epsilon$. As we are not able to accurately estimate the typical sizes of $\bar{A}$ and $\bar{B}$, we instead consider the distinguished asymptotic limit in which they balance the core reaction velocities over the same timescale: from pyruvate to acetyl-CoA, from acetyl-CoA to malonyl-CoA, from malonyl-CoA to malonic semialdehyde, and from malonic semialdehyde to 3HP. The reaction velocity from pyruvate to acetyl-CoA has asymptotic size $O (\min (1,S_1,\epsilon /S_2))$. The reaction velocity from acetyl-CoA to malonyl-CoA has asymptotic size $O (\min (\epsilon ,S_2))$. Balancing these two reaction velocities, and noting that we start with $S_1= O (1)$, we may deduce that the size of these reaction velocities must be of $O (\epsilon )$, and hence we obtain the scaling $S_2= O (1)$. For these reaction velocities to balance with $\bar{A}S_2$, we must consider the distinguished limit $\bar{A}= O (\epsilon )$. Balancing these terms with the reaction velocity from malonyl-CoA to malonic semialdehyde, with asymptotic size $O (\min (1,S_3/\epsilon ))$, we obtain the scaling $S_3= O (\epsilon ^2)$. Then, the balance with $\bar{B}S_3$ yields the distinguished limit $\bar{B}= O (1/\epsilon )$. Therefore, we use the scalings $\bar{A}=\epsilon \hat{A}$ and $\bar{B}= \hat{B}/ \epsilon$, where $\hat{A}, \hat{B}= O (1)$. This appears to be the only distinguished limit of interest.

We now solve this system for two different cases of pyruvate replenishment. The first is continuous pyruvate replenishment, where the pyruvate dynamics are not governed by (3a), and instead we impose $S_1(t) \equiv 1$. The second is no pyruvate replenishment, and (3a) does hold. We will deduce that the two systems are equivalent for $t= O (1)$, and only diverge when $t= O (1/\epsilon )$. In both cases, we are interested in determining how to maximize 3HP production. For the continuous-replenishment case, we are also interested in trying to minimize the long-time levels of malonic semialdehyde, a toxic intermediate compound, present in the cell over a long timescale. As the no-replenishment-of-pyruvate case eventually tends to no metabolites in the system, the long-time levels of malonic semialdehyde in that case are trivial. We now consider the continuous-pyruvate-replenishment case.

## Continuous Replenishment of Pyruvate

**Fig. 2:**
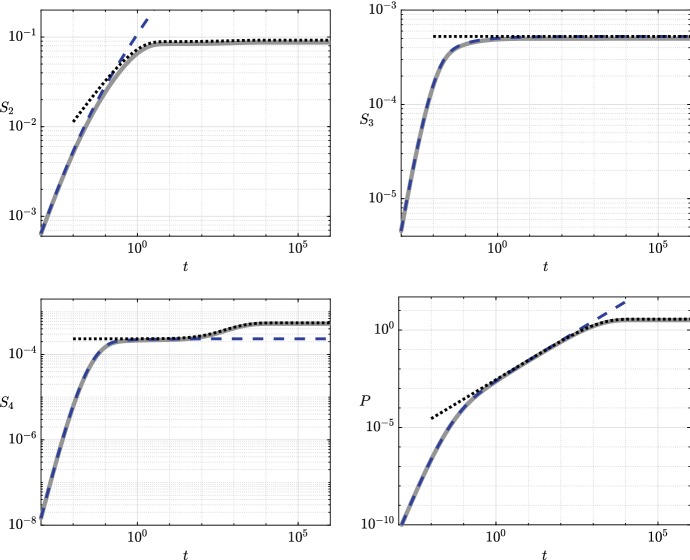
The dynamics of each metabolite in the system when pyruvate is continually replenished. The solid grey lines are the numerical solutions from system (3b–e) with $S_1\equiv 1$, the dashed blue lines are the early-time asymptotic results from (6), and the dotted black lines are the late-time results, where we use the asymptotic results (11), (12), and (15) for $S_3$, $S_4$, and $P$, respectively. These asymptotic results yield a single reduced ODE for $S_2$, given in (9), and it is the numerical solution to this that we plot for $S_2$ as a dotted black line. We use the parameter values given in Table 2 (Color figure online)

We first consider the case where the pyruvate is continuously replenished, modelling the bacteria being harvested in a continuous culture. A numerical simulation of system (3b–e) with $S_1(t) \equiv 1$ shows that the 3HP concentration is bounded above as $t \rightarrow \infty$ (Fig. 2). For our goal of maximizing 3HP production, we would like to understand how to improve the 3HP production by regulating the enzymes in the system. In this section, we solve the nonlinear system (3b–e) with $S_1(t) \equiv 1$ through an asymptotic analysis, exploiting the small parameter $\epsilon$. Our analysis reveals that there is a bifurcation in the asymptotic structure for the large-time behaviour of 3HP with important implications for 3HP production. Our results allow us to quantify this bifurcation in terms of the system parameters, providing physical insight and suggesting which reactions should be the key targets for genetic manipulation.

### Asymptotic Structure

We now discuss the asymptotic structure of the continuous-replenishment case, with reference to the metabolic network shown in Fig. 1. In this case, there are two important timescales in the system: early time, where $t= O (\epsilon )$, and late time, where $t= O (1/\epsilon )$. Over the early timescale, the levels of malonyl-CoA and malonic semialdehyde reach a steady and quasi-steady state, respectively. The remaining metabolite concentrations increase through this early timescale, with a change in the power law governing their growth. Over the late timescale, the levels of acetyl-CoA reach a steady state, but the behaviour of the 3HP depends on the system parameters. There is a bifurcation in the asymptotic structure for the large-time behaviour of 3HP, the levels of which are bounded above unless some critical parameter ratio is reached. We investigate this bifurcation when we consider the late-time behaviour.

### Early Time: $t= O (\epsilon )$

We start our analysis over the distinguished timescale $t= O (\epsilon )$, and thus, we make the scaling $t= \epsilon \hat{t}$. By balancing reaction velocities, we find that the appropriate scalings for the dependent variables over this timescale are $(S_2,S_3, S_4,P) = (\epsilon \hat{S}_2, \epsilon ^2 \hat{S}_3, \epsilon ^2 \hat{S}_4, \epsilon ^2 \hat{P})$, which converts system (3b–e) into

4a $$\begin{aligned} \frac{\mathrm {d} \hat{S}_2}{\mathrm {d} \hat{t}}&= \dfrac{1}{ 1 + \bar{K}_1^M+ \hat{S}_2} - \dfrac{\epsilon \bar{k}_2\hat{S}_2}{\hat{S}_2+ \bar{K}_{2}^M} + \dfrac{\epsilon \bar{k}_{5}\hat{S}_4}{\epsilon \hat{S}_4+ \bar{K}_{5}^M} - \epsilon ^2 \hat{A}\hat{S}_2, \end{aligned}$$

4b $$\begin{aligned} \frac{\mathrm {d} \hat{S}_3}{\mathrm {d} \hat{t}}&= \dfrac{ \bar{k}_2\hat{S}_2}{\hat{S}_2+ \bar{K}_{2}^M} - \dfrac{\bar{k}_{3}\hat{S}_3}{\epsilon \hat{S}_3+ \bar{K}_{3}^M} - \hat{B}\hat{S}_3, \end{aligned}$$

4c $$\begin{aligned} \frac{\mathrm {d} \hat{S}_4}{\mathrm {d} \hat{t}}&= \dfrac{ \bar{k}_{3}\hat{S}_3}{\epsilon \hat{S}_3+ \bar{K}_{3}^M} - \dfrac{\bar{k}_4\hat{S}_4}{\epsilon \hat{S}_4+ \bar{K}_4^M} + \dfrac{\epsilon ^2 \bar{k}_{-4}\hat{P}}{\epsilon ^2 \hat{P}+ \bar{K}_{-4}^M} - \dfrac{\bar{k}_{5}\hat{S}_4}{\epsilon \hat{S}_4+ \bar{K}_{5}^M}, \end{aligned}$$

4d $$\begin{aligned} \frac{\mathrm {d} \hat{P}}{\mathrm {d} \hat{t}}&= \dfrac{ \bar{k}_4\hat{S}_4}{\epsilon \hat{S}_4+ \bar{K}_4^M} - \dfrac{\epsilon ^2 \bar{k}_{-4}\hat{P}}{\epsilon ^2 \hat{P}+ \bar{K}_{-4}^M}. \end{aligned}$$

The leading-order version of (4) is

5 $$\begin{aligned} \frac{\mathrm {d} \hat{S}_2}{\mathrm {d} \hat{t}}&= \dfrac{1}{ 1 + \bar{K}_1^M+ \hat{S}_2}, \quad \frac{\mathrm {d} \hat{S}_3}{\mathrm {d} \hat{t}} = \dfrac{ \bar{k}_2\hat{S}_2}{\hat{S}_2+ \bar{K}_{2}^M} - \left( \bar{v}_3+ \hat{B}\right) \hat{S}_3, \nonumber \\ \frac{\mathrm {d} \hat{S}_4}{\mathrm {d} \hat{t}}&= \bar{v}_3\hat{S}_3- (\bar{v}_4+ \bar{v}_5) \hat{S}_4, \quad \frac{\mathrm {d} \hat{P}}{\mathrm {d} \hat{t}} = \bar{v}_4\hat{S}_4, \end{aligned}$$

where $v_i = k_i/ K_{i}^M$ for $i \in \{3,4,-4, 5\}$. We may solve (5) to obtain

6a $$\begin{aligned} \hat{S}_2(\hat{t})&= \left( 2 \hat{t}+ \left( 1 + \bar{K}_1^M\right) ^2 \right) ^{1/2} - \left( 1 + \bar{K}_1^M\right) , \end{aligned}$$

6b $$\begin{aligned} \hat{S}_3(\hat{t})&= \bar{k}_2\int _0^{\hat{t}} \! \dfrac{ \hat{S}_2(s)}{\hat{S}_2(s) + \bar{K}_{2}^M} \hbox {e}^{(\bar{v}_3+ \hat{B}) (s - \hat{t})} \, \mathrm {d}s, \end{aligned}$$

6c $$\begin{aligned} \hat{S}_4(\hat{t})&= \dfrac{\bar{k}_2\bar{v}_3}{\bar{v}_3+ \hat{B}- \bar{v}_4- \bar{v}_5} \int _0^{\hat{t}} \! \dfrac{ \hat{S}_2(s)}{\hat{S}_2(s) + \bar{K}_{2}^M} \nonumber \\&\quad \left( \hbox {e}^{(\bar{v}_4+ \bar{v}_5) (s - \hat{t})} - \hbox {e}^{(\bar{v}_3+ \hat{B}) (s - \hat{t})} \right) \, \mathrm {d}s, \end{aligned}$$

6d $$\begin{aligned} \hat{P}(\hat{t})&= \dfrac{\bar{k}_2\bar{v}_3\bar{v}_4}{\bar{v}_3+ \hat{B}- \bar{v}_4- \bar{v}_5} \int _0^{\hat{t}} \! \dfrac{ \hat{S}_2(s)}{\hat{S}_2(s) + \bar{K}_{2}^M}\nonumber \\&\quad \left( \dfrac{1 - \hbox {e}^{(\bar{v}_4+ \bar{v}_5) (s - \hat{t})}}{\bar{v}_4+ \bar{v}_5} - \dfrac{1 - \hbox {e}^{(\bar{v}_3+ \hat{B}) (s - \hat{t})}}{\bar{v}_3+ \hat{B}} \right) \, \mathrm {d}s. \end{aligned}$$

We see that the early-time results (6) (dashed black lines) agree very well with the numerical results (solid grey lines) when $t = O (\epsilon ) = O (10^{-2})$ in Fig. 2. However, the metabolite concentrations stray from these asymptotic results (with the exception of $\hat{S}_3$) as *t* becomes larger than of $O (\epsilon )$. This is because new terms in the system become of leading order, and so we must explore a new asymptotic region to understand the system fully. We investigate this in the next section. As such, it is helpful to state the large-$\hat{t}$ limits of solutions (6), as these will allow us to match appropriately into the next timescale. In this limit, we obtain the following expressions

7 $$\begin{aligned}&\hat{S}_2\sim \sqrt{2 \hat{t}}, \quad \hat{S}_3\sim \dfrac{\bar{k}_2}{\bar{v}_3+ \hat{B}}, \quad \hat{S}_4\sim \dfrac{\bar{k}_2\bar{v}_3}{\left( \bar{v}_4+ \bar{v}_5\right) \left( \bar{v}_3+ \hat{B}\right) },\nonumber \\&\hat{P}\sim \dfrac{\bar{k}_2\bar{v}_3\bar{v}_4\hat{t}}{\left( \bar{v}_4+ \bar{v}_5\right) \left( \bar{v}_3+ \hat{B}\right) } \quad \text {as } \hat{t}\rightarrow \infty , \end{aligned}$$

and hence we see that $\hat{S}_3$ and $\hat{S}_4$ reach a steady state over $t= O (\epsilon )$, while $\hat{S}_2$ and $\hat{P}$ continue to grow over the same timescale. In the next subsection, we consider the remaining important timescale $t= O (1/\epsilon )$.

### Late Time: $t= O (1/\epsilon )$

The second and final distinguished timescale for continuous replenishment is $$t= O (1/\epsilon )$$, so we now use the scaling $$t= T/ \epsilon $$. This is the timescale over which there is a balance between the source from pyruvate and the sinks from acetyl-CoA and malonyl-CoA. Over this timescale, $$S_2$$ and $$P$$ are both of $$ O (1)$$, and the scalings for the remaining metabolites are still $$(S_3, S_4) = (\epsilon ^2 \hat{S}_3, \epsilon ^2 \hat{S}_4)$$, which follow from (7), the large-time limits of the early-time solutions. Using the scalings mentioned above, we obtain the late-time version of (3b–e):

8a $$\begin{aligned} \frac{\mathrm {d} S_2}{\mathrm {d} T}&= \dfrac{ 1}{\epsilon \left( 1 + \bar{K}_1^M\right) + S_2} - \dfrac{ \bar{k}_2S_2}{S_2+ \epsilon \bar{K}_{2}^M} + \dfrac{\bar{k}_{5}\hat{S}_4}{\epsilon \hat{S}_4+ \bar{K}_{5}^M} - \hat{A}S_2, \end{aligned}$$

8b $$\begin{aligned} \epsilon ^2 \frac{\mathrm {d} \hat{S}_3}{\mathrm {d} T}&= \dfrac{ \bar{k}_2S_2}{S_2+ \epsilon \bar{K}_{2}^M} - \dfrac{ \bar{k}_{3}\hat{S}_3}{\epsilon \hat{S}_3+ \bar{K}_{3}^M} - \hat{B}\hat{S}_3, \end{aligned}$$

8c $$\begin{aligned} \epsilon ^2 \frac{\mathrm {d} \hat{S}_4}{\mathrm {d} T}&= \dfrac{\bar{k}_{3}\hat{S}_3}{\epsilon \hat{S}_3+ \bar{K}_{3}^M} - \dfrac{ \bar{k}_4\hat{S}_4}{\epsilon \hat{S}_4+ \bar{K}_4^M} + \dfrac{ \bar{k}_{-4}P}{ P+ \bar{K}_{-4}^M} - \dfrac{\bar{k}_{5}\hat{S}_4}{\epsilon \hat{S}_4+ \bar{K}_{5}^M}, \end{aligned}$$

8d $$\begin{aligned} \frac{\mathrm {d} P}{\mathrm {d} T}&= \dfrac{ \bar{k}_4\hat{S}_4}{\epsilon \hat{S}_4+ \bar{K}_4^M} - \dfrac{ \bar{k}_{-4}P}{ P+ \bar{K}_{-4}^M}. \end{aligned}$$

From (8), we obtain the leading-order differential–algebraic system, given by

9a $$\begin{aligned} \frac{\mathrm {d} S_2}{\mathrm {d} T} = \dfrac{1}{S_2} - \bar{k}_2+ \bar{v}_5\tilde{S}_4- \hat{A}S_2, \end{aligned}$$

9b $$\begin{aligned} \frac{\mathrm {d} P}{\mathrm {d} T} = \bar{v}_4\hat{S}_4- \dfrac{ \bar{k}_{-4}P}{ P+ \bar{K}_{-4}^M}, \end{aligned}$$

9c $$\begin{aligned} 0 = \bar{k}_2- \left( \bar{v}_3+ \hat{B}\right) \tilde{S}_3, \end{aligned}$$

9d $$\begin{aligned} 0 = \bar{v}_3\hat{S}_3- \bar{v}_4\hat{S}_4+ \dfrac{ \bar{k}_{-4}P}{ P+ \bar{K}_{-4}^M} - \bar{v}_5\hat{S}_4. \end{aligned}$$

The “initial” conditions of the system are obtained by matching with the large-time results of the early-time system (7), to yield

10 $$\begin{aligned} S_2(0) = 0,\quad \hat{S}_3(0) = \dfrac{\bar{k}_2}{\bar{v}_3+ \hat{B}}, \quad \hat{S}_4(0) = \dfrac{\bar{k}_2\bar{v}_3}{\left( \bar{v}_4+ \bar{v}_5\right) \left( \bar{v}_3+ \hat{B}\right) }, \quad P(0) = 0. \end{aligned}$$

From (9c), we can immediately deduce that $\tilde{S}_3$ is constant over this timescale. That is, over the late timescale, $\tilde{S}_3$ takes the constant value

11 $$\begin{aligned} \tilde{S}_3= \dfrac{\bar{k}_2}{\bar{v}_3+ \hat{B}}. \end{aligned}$$

From (9)–(11), we can write $\tilde{S}_4$ in terms of $P$ as follows:

12 $$\begin{aligned} \tilde{S}_4= \dfrac{1}{\bar{v}_4+\bar{v}_5} \left( \dfrac{\bar{k}_2\bar{v}_3}{\bar{v}_3+ \hat{B}} + \dfrac{\bar{k}_{-4}P}{P+ \bar{K}_{-4}^M} \right) . \end{aligned}$$

Substituting (12) into (9), we obtain the following closed ODE for $P(T)$:

13 $$\begin{aligned} \frac{\mathrm {d} P}{\mathrm {d} T} = \dfrac{\bar{k}_{-4}\bar{v}_5}{\bar{v}_4+\bar{v}_5} \left( \dfrac{\bar{k}_4}{\bar{k}^*} - \dfrac{P}{ P+ \bar{K}_{-4}^M} \right) , \end{aligned}$$

where the critical parameter $\bar{k}^*$ is defined as

14 $$\begin{aligned} \bar{k}^*= \dfrac{\bar{K}_4^M\bar{k}_{-4}\bar{v}_5\left( \bar{v}_3+ \hat{B}\right) }{\bar{k}_2\bar{v}_3}, \end{aligned}$$

and we shall justify shortly the critical nature of $\bar{k}^*$. The differential equation (13) is solved implicitly by

15 $$\begin{aligned} \dfrac{\bar{k}_{-4}\bar{v}_5\left( \bar{k}_4- \bar{k}^*\right) ^2}{\bar{k}^*\left( \bar{v}_4+ \bar{v}_5\right) } T=\left( \bar{k}_4- \bar{k}^*\right) P- \bar{k}^*\bar{K}_{-4}^M\log \left( 1 + \dfrac{\bar{k}_4- \bar{k}^*}{\bar{k}_4\bar{K}_{-4}^M} P\right) . \end{aligned}$$

The asymptotic late-time results (11), (12), and (15) (dotted black lines) agree well with the numerical results (solid grey lines) when $t = O (1/\epsilon ) = O (10^{2})$ in Fig. 2. The remaining nonlinear differential equation (9a) decouples from the rest of the system, and solving this equation is not required to understand the dynamics of the rest of the system. However, we note that the remaining equation will be important when there is no replenishment of pyruvate, which we consider in the next section. To understand the dynamics of $S_2$, (9a) must be solved numerically, and the solution to this agrees well with the full numerical solution of $S_2$ in Fig. 2.

Given that the argument of the logarithm in (15) will vanish for some positive value of $P$ if and only if $\bar{k}_4< \bar{k}^*$, we may deduce that the large-time behaviour of $P$ has a critical dependence on the sign of $\bar{k}_4- \bar{k}^*$. Thus, $\bar{k}^*$ is the bifurcation point for the parameter $\bar{k}_4$, in terms of changing the asymptotic structure of the solution. Specifically, when $\bar{k}_4< \bar{k}^*$, corresponding to a weak reaction from malonic semialdehyde to 3HP, we obtain the bounded large-time behaviour

16a $$\begin{aligned} P\rightarrow \dfrac{\bar{k}_4\bar{K}_{-4}^M}{\bar{k}^*- \bar{k}_4} \quad \text {as } T\rightarrow \infty ; \end{aligned}$$

however when $\bar{k}_4> \bar{k}^*$, corresponding to a strong reaction from malonic semialdehyde to 3HP, we obtain the unbounded large-time behaviour

16b $$\begin{aligned} P\sim \dfrac{\bar{k}_2\bar{v}_3\left( \bar{k}_4- \bar{k}^*\right) }{\bar{K}_4^M\left( \bar{v}_3+ \hat{B}\right) \left( \bar{v}_4+ \bar{v}_5\right) } T\quad \text {as } T\rightarrow \infty . \end{aligned}$$

**Fig. 3:**
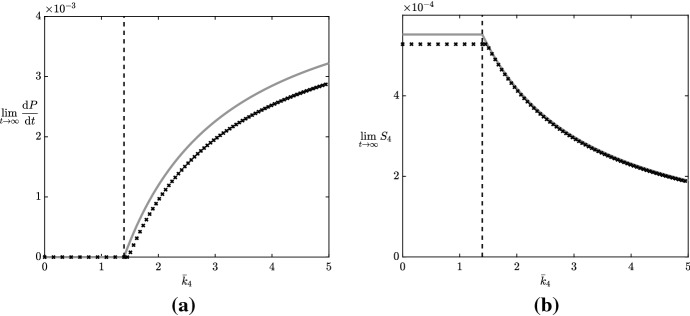
The large-time limits of **a** 3HP production and **b** malonic semialdehyde varying over the critical parameter $\bar{k}_4= \bar{k}^*$. The grey lines denote the asymptotic predictions [from (16) and (18)], and the black crosses denote numerical results (solving (3b–e) with $S_1\equiv 1$). The subcritical and supercritical regimes are to the left and right, respectively, of the dashed lines in each subfigure. To obtain the large-time numerical limits, we run the simulations until $t = t_\text {end} := 10^{12}$; we approximate $\lim _{t \rightarrow \infty } \mathrm {d}P/\mathrm {d}t \approx P(t_\text {end})/t_\text {end}$ and $\lim _{t \rightarrow \infty } S_4 \approx S_4(t_\text {end})$. Apart from $\bar{k}_4$, which we vary in this figure, we use the parameter values given in Table 2, and these correspond to an asymptotic value of the critical parameter $\bar{k}^*\approx 1.3951$

We show this bifurcation behaviour in Fig. 3a, including a comparison between the numerical and asymptotic results. We see that our numerical results show the bifurcation behaviour predicted by our asymptotic results. Moreover, our asymptotic results are good predictions of both the location of the bifurcation and the long-time behaviour.

It is of mathematical interest to briefly note that when $\bar{k}_4= \bar{k}^*$, the solution to (13) is

17 $$\begin{aligned} P= \bar{K}_{-4}^M\left[ \left( 1 + \dfrac{2 \bar{v}_{-4}\bar{v}_5T}{\bar{v}_4+ \bar{v}_5} \right) ^{1/2} - 1\right] \quad \text {as } T\rightarrow \infty . \end{aligned}$$

Hence, as $\bar{k}_4$ passes through the bifurcation point, the long-time behaviour of $P$ is to increase with the square root of time.

From (12), we see that $\tilde{S}_4$ will tend to a constant in the far-field; for $\bar{k}_4< \bar{k}^*$, we have the subcritical regime

18a $$\begin{aligned} \tilde{S}_4\rightarrow \dfrac{\bar{k}_2\bar{v}_3}{\bar{v}_5\left( \bar{v}_3+ \hat{B}\right) } \quad \text {as } T\rightarrow \infty , \end{aligned}$$

and for $\bar{k}_4> \bar{k}^*$, we have the supercritical regime

18b $$\begin{aligned} \tilde{S}_4\rightarrow \dfrac{1}{\bar{v}_4+ \bar{v}_5} \left( \dfrac{\bar{k}_2\bar{v}_3}{\bar{v}_3+ \hat{B}} + \bar{k}_{-4}\right) \quad \text {as } T\rightarrow \infty . \end{aligned}$$

We show this bifurcating behaviour in Fig. 3b, including a comparison between the numerical and asymptotic results. As before, we see that our numerical results display the bifurcation behaviour predicted by our asymptotic results, and that the latter are a good predictor of the bifurcation behaviour. Recall that our goal for the continuous-pyruvate-replenishment case is to maximize 3HP production while minimizing the large-time levels of malonic semialdehyde, the latter being a toxic intermediate compound. Therefore, as large-time 3HP production is zero and malonic semialdehyde levels are higher in the subcritical regime, Fig. 3b tells us immediately that the subcritical regime is a bad regime for industrially viable 3HP production, and that the parameter regime $\bar{k}_4> \bar{k}^*$ is much better for our goal. From Table 2, we note that the critical parameter $\bar{k}^*\approx 1.3951$, but $\bar{k}_4= 0.67$. Therefore, the simulations in Fig. 2 occur in the subcritical regime, as can be deduced by noting that the 3HP concentration is bounded above. Our model predicts that one can achieve large gains in 3HP production if one is able to move into the supercritical regime.

### Physical Implications

To frame our discussion around parameters that can be varied experimentally (essentially the levels of each enzyme, regulated by the over- or under-expression of the genes that control their production), we rewrite our results in terms of dimensional quantities. In dimensional terms, the bifurcation occurs when $k_4= k^*$, where

19 $$\begin{aligned} k^*= \dfrac{k_{-4}k_{5}K_4^ME_{5}}{k_2K_{5}^ME_2} \left( 1 + \dfrac{BK_{3}^M}{k_{3}E_{3}} \right) . \end{aligned}$$

From (19), we see that an up-regulation of $E_2$ and a down-regulation of $E_{5}$ have the most significant effect on lowering $k^*$, the critical bifurcation parameter, and thus hopefully moving into the supercritical regime for significantly increased 3HP production. Additionally, an up-regulation of $E_{3}$ will also have an effect in lowering $k^*$, but with diminishing returns. Notably, our model suggests that regulation of neither $E_1$ nor $E_4$ has a significant effect on this critical bifurcation parameter (see below for a potential important role for $E_4$, however).

The supercritical regime occurs when $k_4> k^*$, and this corresponds to a nonzero large-time production of 3HP, which is the target if we are to attain the goal of industrially viable 3HP production. In this case, the dimensional large-time production of 3HP is

20 $$\begin{aligned} \frac{\mathrm {d} [P]}{\mathrm {d} \tau } \sim \dfrac{k_2E_2\left( 1 - \dfrac{k_{-4}k_{5}K_4^ME_{5}}{k_2k_4K_{5}^ME_2} \left( 1 + \dfrac{BK_{3}^M}{k_{3}E_{3}}\right) \right) }{\left( 1 + \dfrac{BK_{3}^M}{k_{3}E_{3}} \right) \left( 1 + \dfrac{k_{5}K_4^ME_{5}}{k_4K_{5}^ME_4} \right) }, \end{aligned}$$

and the dimensional large-time levels of malonic semialdehyde in the system are

21 $$\begin{aligned}{}[S_4]\sim \dfrac{k_2K_4^ME_2\left( 1 + \dfrac{k_{-4}E_4}{k_2E_2} \left( 1 + \dfrac{BK_{3}^M}{k_{3}E_{3}}\right) \right) }{ k_4E_4\left( 1 + \dfrac{BK_{3}^M}{k_{3}E_{3}}\right) \left( 1 + \dfrac{k_{5}K_4^ME_{5}}{k_4K_{5}^ME_4} \right) }. \end{aligned}$$

As our goal for the continuous-replenishment case is to maximize 3HP production while minimizing the large-time levels of malonic semialdehyde, we consider the ratio of the large-time levels of $\mathrm {d}[P]/\mathrm {d}\tau$ to $[S_4]$, to obtain

22 $$\begin{aligned} \dfrac{\frac{\mathrm {d} [P]}{\mathrm {d} \tau }}{[S_4]} \sim \dfrac{ k_2k_4E_2E_4\left( 1 - \dfrac{k_{-4}k_{5}K_4^ME_{5}}{k_2k_4K_{5}^ME_2} \left( 1 + \dfrac{BK_{3}^M}{k_{3}E_{3}}\right) \right) }{ K_4^M\left( k_2E_2+ k_{-4}E_4\left( 1 + \dfrac{BK_{3}^M}{k_{3}E_{3}}\right) \right) }. \end{aligned}$$

**Fig. 4:**
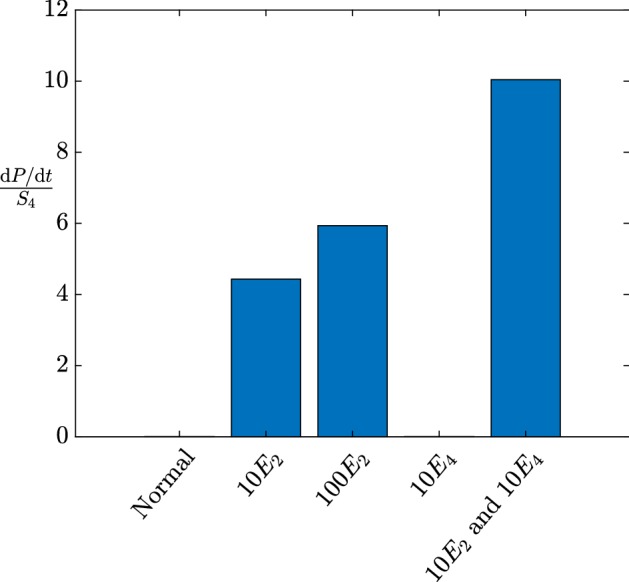
The effect of regulating enzymes in the pathway shown in Fig. 1 on the key ratio (22), obtained by solving (3b–e) with $S_1\equiv 1$. We use the parameter values in Table 2, but with increased enzyme concentrations as specified on the *x*-axis. While up-regulating $E_2$ yields improvement, this has diminishing returns. However, even though up-regulating $E_4$ by itself does not appear to have a significant effect on the ratio, up-regulating $E_2$ and $E_4$ in tandem does have a significant effect (Color figure online)

While (22) appears to have a convoluted form, it provides a quantitative value that we wish to maximize. In particular, we are able to see that the most significant gains can be made by up-regulating $E_2$ and $E_4$ in tandem. Our model suggests that an increase in only one of these enzymes will increase ratio (22), but with diminishing returns (Fig. 4). However, increasing both enzymes will result in significant increases in the 3HP production to malonic semialdehyde ratio (Fig. 4). Additionally, we note that 3HP production can also be increased by up-regulating $E_{3}$ and down-regulating $E_{5}$, though this will have diminishing returns. We emphasize that maximizing (22) should not be considered a definitive metric, since the toxic effect of malonic semialdehyde ($[S_4]$) will have its own nonlinear effects for which to account. Other relevant metrics could involve keeping the maximum value of malonic semialdehyde below a critical value or keeping the cumulative presence of malonic semialdehyde low. Our multiscale approach allows for the efficient analysis of any such metric.

## No Replenishment of Pyruvate

We now consider the case where the pyruvate is never replenished. Due to the connectivity of the metabolic network and the sinks in the system, this problem results in a long-term decay of each metabolite. As the only metabolite initially present in the system is pyruvate, the remaining metabolites will each reach a maximum level at some (generally different) point in time. Our main goal in this subsection is to obtain an asymptotic expression for the maximum level of 3HP present in the system, and the time at which this occurs. In a batch culture system, where nothing is added or removed to the system until the run is stopped for harvesting, this time provides an approximation for when to stop the run and harvest the product. We will summarize the system that governs this optimal time and product yield, but we relegate the details to “Appendix A” for brevity.

### Asymptotic Structure

**Fig. 5:**
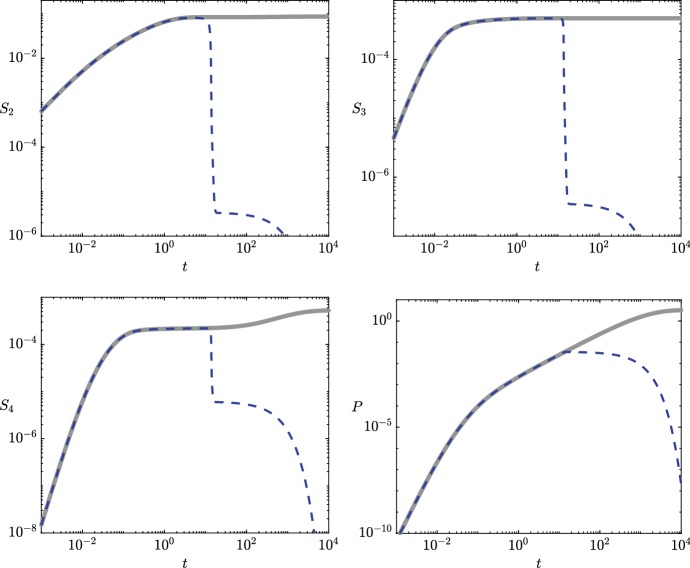
A comparison between the metabolic dynamics in the continuous- and no-replenishment-of-pyruvate cases. The solid grey lines are the numerical solutions from the continuous-replenishment-of-pyruvate system (3b–e) with $S_1\equiv 1$, and the dashed blue lines are the numerical solutions from the no-replenishment-of-pyruvate system (3). We see that the two cases are essentially equivalent until around $t = 5$, after which the metabolite levels drop in a sharp manner for $S_2$, $S_3$, and $S_4$, and at a slower rate for *P*. The maximal level of 3HP occurs around the time of the sharp drop. We use the parameter values given in Table 2 for these figures (Color figure online)

We now discuss the asymptotic structure of the no-replenishment case, with reference to the metabolic network shown in Fig. 1. The no-replenishment-of-pyruvate case is equivalent to the continuous-replenishment case at leading order until we reach the timescale $t= O (1/\epsilon )$ (as shown in Fig. 5 for $S_2$, $S_3$, $S_4$, and *P*), at which point the depletion of pyruvate occurs as a leading-order effect.

**Fig. 6:**
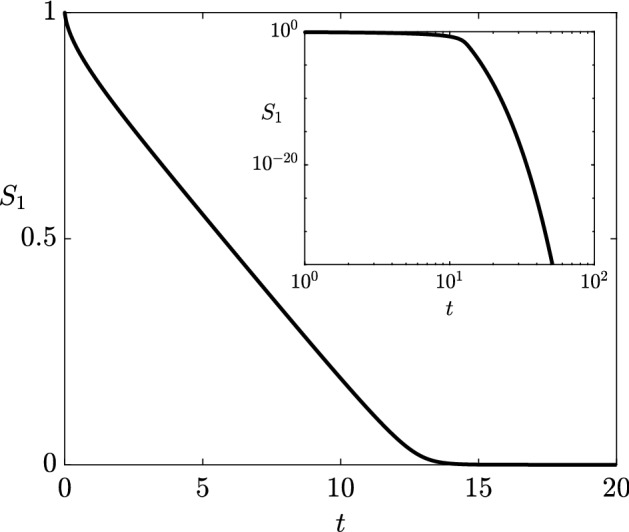
The dynamics of pyruvate ($S_1$) when it is never replenished, obtained from solving (3). We see that it becomes exponentially small shortly after $t = 12$. We use the parameter values given in Table 2

Over this longer timescale, the rest of the system remains unchanged until the levels of pyruvate are of $O (\epsilon )$, which we define to occur at $t= T_d/\epsilon = O (1/\epsilon )$. The pyruvate is then depleted to a negligible level at a relatively quicker rate, over a timescale of $t- T_d/\epsilon = O (1)$, as can be seen in Fig. 6, while the remaining metabolites are unchanged at leading order. After this rapid depletion of pyruvate, the reaction from pyruvate to acetyl-CoA is negligible and so the levels of acetyl-CoA start to deplete, again over the timescale of $t= O (1/\epsilon )$. When the levels of acetyl-CoA become of $O (\epsilon )$, which we define to occur at $t= T^*/\epsilon = O (1/\epsilon )$, the Michaelis–Menten reaction from acetyl-CoA to malonyl-CoA becomes unsaturated, and the lower levels of acetyl-CoA are felt through the rest of the system. After this point, all the remaining metabolites start to deplete over a timescale of $t= O (1/\epsilon )$, and so $t= T^*/\epsilon$ marks the time at which the level of 3HP in the system is maximal. In the next section, we present the reduced system required to be solved to determine $T^*$.

### Reduced System for $T^*$

The time at which the level of 3HP in the system is maximal is given by $t= T^*/\epsilon + O (1)$, where $T^*= O (1)$. To determine $T^*$ at leading order, it suffices to solve the reduced system

23a $$\begin{aligned} \frac{\mathrm {d} S_2}{\mathrm {d} T}&= f(S_2) - \bar{k}_2+ \bar{v}_5\hat{S}_4(T) - \hat{A}S_2(T) \nonumber \\&\quad \text {for } 0< T< T^*, \quad \text {with } S_2\sim \left( 2 T\right) ^{1/2} \text { as } T\rightarrow 0^{+}, \end{aligned}$$

and we provide a derivation of this reduced system in “Appendix A”. In (23a), the nonlinear function *f* is given by

23b $$\begin{aligned} f(S_2) = {\left\{ \begin{array}{ll} \dfrac{1}{S_2} &{}\text{ if } 0< T< T_d, \\ 0 &{} \text{ if } T_d< T< T^*. \end{array}\right. } \end{aligned}$$

Additionally, since the remaining metabolite concentrations are equivalent to those in the continuous-replenishment case until acetyl-CoA depletion occurs, the concentration of malonic semialdehyde $\hat{S}_4$ is given by the implicit representation (12), (14)–(15). As shown in “Appendix A”, the asymptotic leading-order time at which pyruvate depletion occurs is $T= T_d$, where $T_d$ satisfies the implicit equation

23c $$\begin{aligned} \int _0^{T_d} \! f(S_2(s)) \, \mathrm {d} s = 1. \end{aligned}$$

The time at which there is a maximal amount of 3HP in the system $T= T^*$ is when acetyl-CoA depletion occurs, since this is when the remaining metabolites in the system start to feel the lack of the previous metabolites. Asymptotically, this occurs when $S_2(T^*) = 0$ or, equivalently, for $T^*$ satisfying the implicit equation

23d $$\begin{aligned} 1 + \bar{v}_5\int _0^{T^*} \! \hat{S}_4(s) \, \mathrm {d} s = \bar{k}_2T^*+ \hat{A}\int _0^{T^*} \! S_2(s) \, \mathrm {d} s, \end{aligned}$$

which is obtained by integrating (23a) in time and using (23c). It is a simple task to solve this system numerically for any specified parameter values. Then, the maximal amount of 3HP in the system can be obtained by substituting $T^*$ into (15) to yield the implicit equation

24 $$\begin{aligned} \dfrac{\bar{k}_{-4}\bar{v}_5\left( \bar{k}_4- \bar{k}^*\right) ^2}{\bar{k}^*\left( \bar{v}_4+ \bar{v}_5\right) } T^*=\left( \bar{k}_4- \bar{k}^*\right) P(T^*) - \bar{k}^*\bar{K}_{-4}^M\log \left( 1 + \dfrac{\bar{k}_4- \bar{k}^*}{\bar{k}_4\bar{K}_{-4}^M} P(T^*) \right) . \end{aligned}$$

**Fig. 7:**
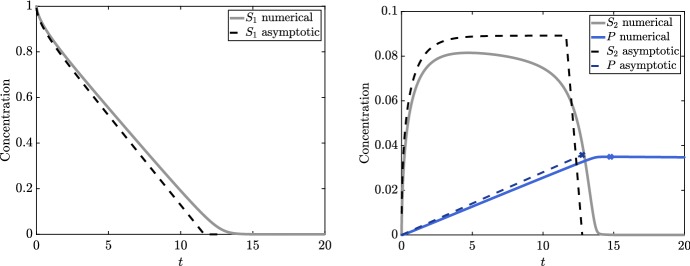
A comparison of numerical solutions for the full system in (3) and the reduced system presented in Sect. 4.2. Our solutions for the reduced system are valid for $0< t < T^*/\epsilon$. Here, $t = T^*/\epsilon$ is the asymptotic result for the point at which the 3HP in the system is maximal, and $P(T^*)$ in (24) gives the asymptotic result for the maximal level of 3HP. We mark the maximal value of 3HP in the system with a cross in the appropriate colour for the numerical and asymptotic solutions, respectively. We use the parameter values given in Table 2 (Color figure online)

**Fig. 8:**
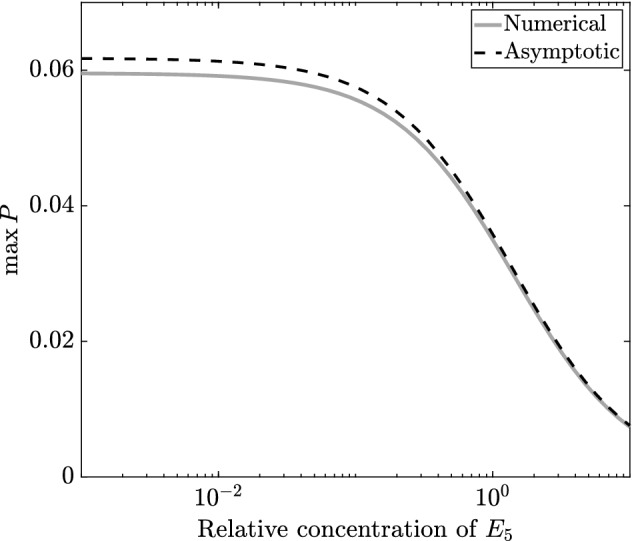
The maximum amount of 3HP in the system as the concentration of $E_{5}$ is varied, for the full numerical system (3) and the reduced system presented in Sect. 4.2. We use parameter values from Table 2, with $E_{5}$ modified as specified on the *x*-axis. The maximum discrepancy between numerical and asymptotic solutions for the maximum value of 3HP is around 4%, which occurs for smaller relative concentrations of $E_{5}$

Comparing this reduced system to the full system in Fig. 7, we see that the maximal 3HP produced is very similar between the asymptotic and numerical results, and that there is an $$ O (1)$$ difference in the predicted time at which this maximal 3HP is produced between the two, as predicted by the error in our asymptotic results. We show this excellent agreement for a range of relative concentrations of $$E_{5}$$ (Fig. 8), the enzyme which recycles malonic semialdehyde back into acetyl-CoA. Moreover, we see that more 3HP is produced when $$E_{5}$$ is down-regulated.

An alternative implicit equation for $T^*$ is given by (45), which uses the analytic solution for $S_2$ when $T_d< T< T^*$, stated in (44).

### Asymptotic Expression for $T^*$ when $\bar{v}_5\rightarrow 0$

Our model predicts that 3HP production can be improved by under-expressing the enzyme that controls the reaction from malonic semialdehyde to acetyl-CoA, which has maximal reaction rate $\bar{k}_{5}$ (Fig. 8). If this reaction is completely removed from the metabolic network, we are able to provide an analytic expression for $T^*$ and $P(T^*)$. This corresponds to taking the limit $\bar{k}_{5}\rightarrow 0$, equivalent to $\bar{v}_5\rightarrow 0$. In this limit, there is no further production or depletion of 3HP when $t> T^*/\epsilon$, and the total amount of 3HP produced is given by $P(T^*)$.

As $\bar{v}_5\rightarrow 0$, there are several scalings that reduce the parameter dependence of the system. We scale $T= \bar{T}/ \hat{A}$ and $S_2(T) = \bar{k}_2Y(\bar{T}) / \hat{A}$, to turn (23a) into

25a $$\begin{aligned} \frac{\mathrm {d} Y}{\mathrm {d} \bar{T}} = \bar{f}(Y) - 1 - Y\quad \text {for } 0< \bar{T}< \bar{T}^*, \quad \text {with } Y\sim \left( 2 \alpha \bar{T}\right) ^{1/2} \text { as } \bar{T}\rightarrow 0^{+}, \end{aligned}$$

where $\alpha = \hat{A}/ \bar{k}_2^2$, $T^*= \bar{T}^*/ \hat{A}$, the nonlinear function $\bar{f}$ is defined as

25b $$\begin{aligned} \bar{f}(Y) = {\left\{ \begin{array}{ll} \dfrac{\alpha }{Y} &{}\text{ if } 0< \bar{T}< \bar{T}_d, \\ 0 &{} \text{ if } \bar{T}_d< \bar{T}< \bar{T}^*, \end{array}\right. } \end{aligned}$$

and $T_d= \bar{T}_d/ \hat{A}$ has the form

25c $$\begin{aligned} \int _0^{\bar{T}_d} \! \dfrac{\mathrm {d} \bar{T}}{Y(\bar{T})} = 2 \beta , \end{aligned}$$

where $\beta = \bar{k}_2/ 2$, which will be useful later for notational purposes.

When $0< \bar{T}< \bar{T}_d$, the system (25a) is solved implicitly by

26 $$\begin{aligned} -2 \bar{T}= \log \left( \dfrac{\alpha - Y- Y^2}{\alpha } \right) + \dfrac{2 }{\sqrt{1 + 4 \alpha }} \tanh ^{-1} \left[ \dfrac{\left( \sqrt{1 + 4 \alpha }\right) Y}{2 \alpha - Y}\right] . \end{aligned}$$

We are able to obtain an explicit expression for $Y(\bar{T}_d)$ by re-writing (25c) as

27 $$\begin{aligned} 2 \beta = \int _0^{Y(\bar{T}_d)} \! \dfrac{\mathrm {d} Y}{Y\dot{Y}} = \int _0^{Y(\bar{T}_d)} \! \dfrac{\mathrm {d} Y}{\alpha - Y- Y^2} = \dfrac{2}{\sqrt{1 + 4 \alpha }} \tanh ^{-1} \left[ \dfrac{\left( \sqrt{1 + 4 \alpha }\right) Y(\bar{T}_d)}{2 \alpha - Y(\bar{T}_d)} \right] , \end{aligned}$$

where we use (25a) for $\dot{Y} \equiv \mathrm {d}Y/\mathrm {d}\bar{T}$. Re-arranging (27), we obtain

28a $$\begin{aligned} Y(\bar{T}_d) = \dfrac{2 \alpha \tanh \left( \beta \sqrt{1 + 4 \alpha }\right) }{\sqrt{1 + 4 \alpha } + \tanh \left( \beta \sqrt{1 + 4 \alpha }\right) }. \end{aligned}$$

We can then obtain an explicit expression for $\bar{T}_d$ by substituting (27)–(28a) into (26), to yield

28b $$\begin{aligned} \bar{T}_d= \log \left[ \dfrac{\sqrt{1 + 4 \alpha } +\tanh \left( \beta \sqrt{1 + 4 \alpha }\right) }{\sqrt{1 + 4 \alpha } {{\,\mathrm{sech}\,}}\left( \beta \sqrt{1 + 4 \alpha }\right) } \right] - \beta . \end{aligned}$$

When $\bar{T}_d< \bar{T}< \bar{T}^*$, system (25a) is solved explicitly by

29a $$\begin{aligned} Y(\bar{T}) = \left( 1 + Y(\bar{T}_d)\right) \hbox {e}^{\bar{T}_d-\bar{T}} - 1, \end{aligned}$$

and as $\bar{T}^*$ satisfies $Y(\bar{T}^*) = 0$, we can state $\bar{T}^*$ in terms of $\bar{T}_d$ as follows:

30 $$\begin{aligned} \bar{T}^*= \bar{T}_d+ \log \left( 1 + Y(\bar{T}_d) \right) , \end{aligned}$$

where each term in (30) can be determined explicitly from (28).

As we provide the general implicit result for maximal 3HP in (24), we can derive a reduced version for the limit we consider in this subsection by taking the same limit, $\bar{v}_5\rightarrow 0$, noting that $\bar{k}^*$ also scales with $\bar{v}_5$. Using (30), the reduced result for the time at which the levels of 3HP are maximal, the total amount of 3HP produced is given explicitly by

31a $$\begin{aligned} P(T^*)&= \left( \dfrac{\bar{v}_3}{\bar{v}_3+ \hat{B}}\right) F(\alpha ,\beta ), \end{aligned}$$

31b $$\begin{aligned} F(\alpha ,\beta )&:= \dfrac{ 1}{2 \alpha \beta }\left( \log \left[ \dfrac{\sqrt{1 + 4 \alpha } +\left( 1 + 2 \alpha \right) \tanh \left( \beta \sqrt{1 + 4 \alpha }\right) }{\left( \sqrt{1 + 4 \alpha } \right) {{\,\mathrm{sech}\,}}\left( \beta \sqrt{1 + 4 \alpha }\right) } \right] - \beta \right) . \end{aligned}$$

**Fig. 9:**
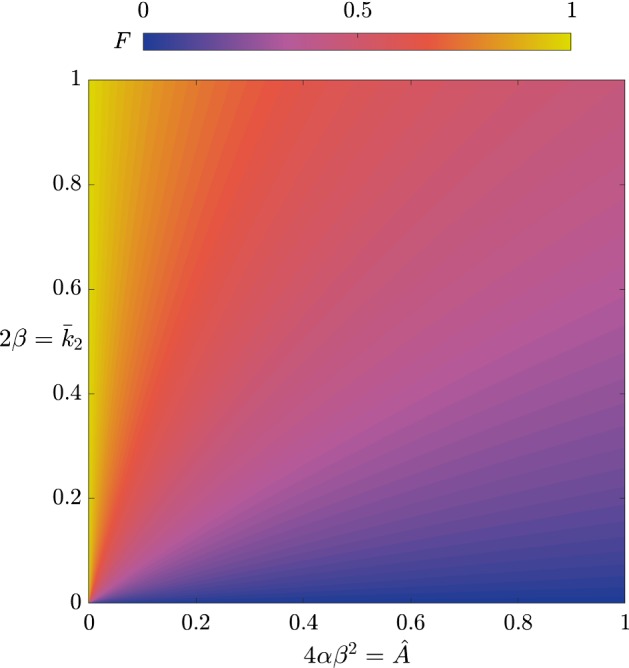
The asymptotic result for $F(\alpha ,\beta )$, the total 3HP produced with no replenishment of pyruvate in the limit of $\bar{v}_5\rightarrow 0$, given in (31). We use axes $4\alpha \beta ^2$ and $2\beta$, which are proxies for the strength of the sink and source in the system, respectively (Color figure online)

To visualize (31), we provide a plot of $F(\alpha ,\beta )$ in Fig. 9, but as a function of $\alpha \beta ^2 = \hat{A}/4$ and $\beta = \bar{k}_2/ 2$, as these are proxies for the strength of the sink and source in the system, respectively. As *F* is bounded above by 1 and this limit is attained as $\alpha \rightarrow 0$, decreasing $\alpha$ (and hence $\hat{A}$) to zero will have a more significant effect than any finite increase in $\beta$ (and hence $\bar{k}_2$), as long as the organism is able to survive with low values of $\hat{A}$.

#### Physical Implications

In a similar manner to the analysis in Sect. 3.4, we now discuss the physical implications of our results from this subsection and we reintroduce dimensional variables to our key results.

The first key result is the time at which the 3HP levels are maximal, $\tau = \tau ^*$, where

32 $$\begin{aligned} \tau ^* = \dfrac{S_0T^*}{k_1E_1\epsilon }. \end{aligned}$$

**Fig. 10:**
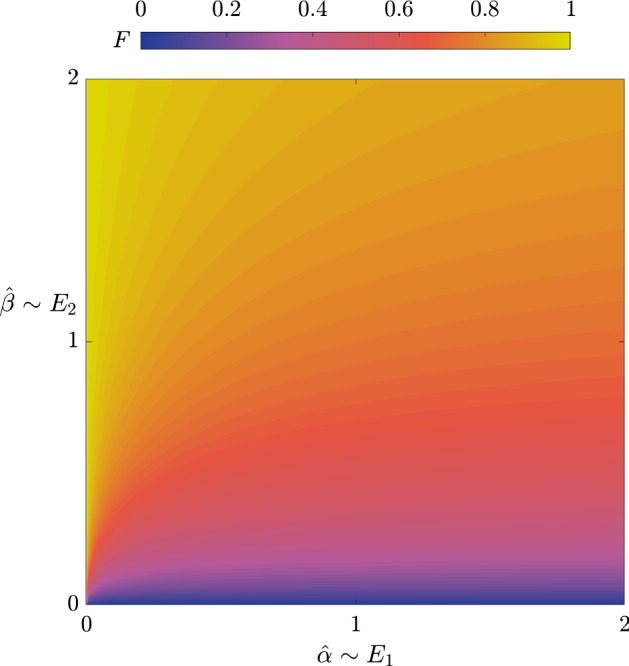
A plot showing $F(\hat{\alpha },\hat{\beta })$, the asymptotic result for the total 3HP produced with no replenishment of pyruvate in the limit of $\bar{v}_5\rightarrow 0$, given in (31). In contrast to Fig. 9, we present *F* in terms of a variation in the parameters $E_1$ and $E_2$ (as shown in (35)), corresponding to regulation of enzymes (Color figure online)

The effective production rate of 3HP is

33 $$\begin{aligned} \dfrac{[P](\tau ^*)}{\tau ^*} = \dfrac{k_2k_{3}E_2E_{3}}{k_{3}E_{3}+ BK_{3}^M}, \end{aligned}$$

which can be increased by increasing $E_2$ or $E_{3}$, though the latter has diminishing returns. The total amount of 3HP produced is given by

34 $$\begin{aligned}{}[P](\tau ^*) = \dfrac{S_0k_{3}E_{3}}{k_{3}E_{3}+ BK_{3}^M} F\left( \dfrac{Ak_1E_1K_1^i}{k_2^2 E_2^2},\dfrac{S_0k_2E_2}{2 K_1^ik_1E_1}\right) , \end{aligned}$$

where *F* is defined in (31). Defining

35 $$\begin{aligned} \hat{\alpha } = \dfrac{1}{\alpha \beta ^2} = \dfrac{4 K_1^ik_1E_1}{AS_0^2}, \quad \hat{\beta } = \dfrac{1}{\alpha \beta } = \dfrac{2 k_2E_2}{AS_0}, \end{aligned}$$

as proxies for $E_1$ and $E_2$, we see that up-regulating $E_2$ results in higher levels of 3HP, whereas up-regulating $E_1$ results in lower levels of 3HP, with significant diminishing returns as $E_1$ increases (Fig. 10). The negative effect of up-regulating $E_1$ may appear counter-intuitive, since $E_1$ is required for a connected pathway. However, it can be explained by noting that the effect of increasing $E_1$ is to increase the amount of acetyl-CoA in the system, which has the result of greatly increasing the amount of acetyl-CoA that is taken up by the sink, but only slightly increasing the amount converted into malonyl-CoA. This is because we have assumed that we are in a regime where the sink has first-order kinetics, whereas the reaction from acetyl-CoA to malonyl-CoA follows Michaelis–Menten-type kinetics. This effect may be reduced if we were in a regime where the acetyl-CoA sink was saturating. Hence, our model suggests that a slow conversion of pyruvate into acetyl-CoA is better for 3HP production if it is not possible to over-regulate $E_2$. We note that this down-regulation is only important for the maximum amount of 3HP produced when the time taken to produce this 3HP is taken into account, as there is no dependence on $E_1$ in the effective production rate (33). We emphasize that $E_1\rightarrow 0$ is a singular limit in the system, which appears because we are in the no-replenishment-of-pyruvate regime. As $E_1\rightarrow 0$, the early-time kinetics of the problem would vary, and we would not have the same late-time problem that we consider here. We do not analyse this limit further, since it is not of particular physical interest.

## Discussion

We develop and solve a mathematical model for the reaction kinetics of the 3-hydroxypropionic acid (3HP) pathway from pyruvate via malonyl-CoA. We consider two main cases, continuous and no replenishment of pyruvate, to model the metabolite dynamics in the two extreme cases of plentiful and scarce pyruvate. Our main goal is to understand how to maximize 3HP production, and in the case of a continuous pyruvate replenishment to additionally minimize the maximum levels of the toxic intermediary malonic semialdehyde in the system, where possible. We summarize our enzyme regulation recommendations for each case in Table 3.

**Table 3:** A summary of the enzyme regulations we recommend based on the results of our model

Enzyme [EC number(s)]	Continuous replenishment	No replenishment
Pyruvate dehydrogenase complex [1.2.4.1, 2.3.1.12, 1.8.1.4]	No effect	Down-regulate
Acetyl-CoA carboxylase [6.4.1.2]	Up-regulate	Up-regulate
Malonyl-CoA reductase [1.2.1.75]	Up-regulate	Up-regulate
3-Hydroxypropionate dehydrogenase [1.1.1.298]	Up-regulate	Up-regulate
Malonate semialdehyde dehydrogenase (acetylating) [1.2.1.18]	Down-regulate	Down-regulate

We give results for the two cases of continuous and no replenishment of pyruvate

These are the extremes of the actual time-dependent pyruvate replenishment in the cell and can model continuous and batch culture, respectively

We note that while our regulation recommendations suffer from diminishing returns if implemented on a single enzyme at a time, we can overcome this issue in the continuous-replenishment-of-pyruvate case if we up-regulate acetyl-CoA carboxylase (EC 6.4.1.2) in tandem with 3-hydroxypropionate dehydrogenase (EC 1.1.1.298). Even though a solo up-regulation of either of these enzymes has diminishing returns on improving the ratio of 3HP production rate to maximum malonic semialdehyde, our analysis demonstrates that the up-regulation of both enzymes at the same time has the nonlinear effect of strongly increasing this ratio with no diminishing returns, as shown in Fig. 4.

We also emphasize that the regulation of pyruvate dehydrogenase complex (EC 1.2.4.1, EC 2.3.1.12, and EC 1.8.1.4) is only important when the pyruvate is scarce. In this case, the total 3HP produced can be increased, perhaps counterintuitively, by a slight down-regulation of pyruvate dehydrogenase complex. This is because there are competing destinations for acetyl-CoA—either a sink out of the system or onwards to malonyl-CoA—and the route out of the system becomes favoured as the amount of acetyl-CoA in the system increases. Hence, a slower production of acetyl-CoA from pyruvate is preferable to channel more acetyl-CoA towards malonyl-CoA rather than to the sink, which means a down-regulation of pyruvate dehydrogenase complex. We note that this effect disappears when we account for the time taken to produce the maximal 3HP; the effective production rate of 3HP in this case has no dependence on pyruvate dehydrogenase complex. Moreover, we emphasize that this down-regulation effect is a singular limit of the system. Hence, one cannot obtain maximal 3HP by completely removing pyruvate dehydrogenase complex from the system—in fact the amount of 3HP produced would be zero if this enzyme were completely removed from the system.

To deal with an inherent uncertainty in the parameter values, we maximize our analytic progress by using an asymptotic analysis to solve the system, exploiting the significant difference in typical parameter ratios. This allows us to systematically understand the effect of up- and down-regulating different enzymes without resorting to an expensive parameter sweep. In the continuous-replenishment-of-pyruvate case, our asymptotic analysis reveals a bifurcation in the asymptotic structure of the problem; we quantify the effect of this bifurcation for 3HP production and derive an analytic expression for the critical surface in parameter space at which this occurs. Physically, this bifurcation corresponds to a sudden change in the large-time 3HP production across the critical parameter.

For reactions between the metabolites we tracked, we use modified Michaelis–Menten reaction velocities obtained from the literature, including the effect of inhibition in the reaction from pyruvate to acetyl-CoA from both of these metabolites. We add the effect of acetyl-CoA and malonyl-CoA loss to other metabolic pathways by including an aggregate sink from these metabolites, governed using first-order reaction kinetics. The initial conditions we used corresponded to an instantaneous introduction of pyruvate to a well-mixed solution of enzymes containing no other metabolites. We choose these conditions for mathematical convenience, as the real cells are likely to contain an unknown level of the other metabolites in the system when production is initiated. We do not expect this to be an issue for the case with a continuous replenishment of pyruvate, in that we expect a small initial amount of intermediate metabolites to change the early-time problem only. However, this is not true for the case with no replenishment of pyruvate, as the depletion dynamics will be sensitive to the initial conditions. Obtaining these measurements for the initial conditions would be difficult *in vivo*, though can be achieved by rapidly quenching the cellular metabolism before using mass spectrometry to examine the metabolites (Bennett et al. 2009). In addition, there may be cases where the assumption of a well-mixed system does not hold, in particular over larger lengthscales, *e*.*g*. a colony of bacteria. In such cases, one could determine effective reaction rates by coupling the nonlinear reaction dynamics we consider here to spatial transport processes over longer lengthscales. This procedure could be carried out in a computationally efficient manner if one exploited the extreme ratios of the different lengthscales in the system, as performed in Dalwadi et al. (2018) for diffusive transport and linear reactions.

In this paper, we have accounted for the toxicity of malonic semialdehyde by constructing appropriate metrics to evaluate our results. That is, the toxic effect is not directly included in our model assumptions, only analysed *post hoc*. Since this toxicity has a negative effect on biomass production, it could be included as a negative feedback in (1) if appropriately quantified.

Finally, we note how this works highlights how mathematical modelling and asymptotic techniques can be used to understand a biological system, and to address the key questions facing experimentalists. In this case, to identify a combination of enzyme regulation with a greater theoretical output than could be obtained by measuring outputs from regulating just one enzyme at a time. This key insight into the system behaviour was possible due to the asymptotic analysis allowing us to overcome uncertainty in parameter values. We hope that these methods can be used to understand other biological systems, and to reduce the time taken to explore their experimental parameter space.

## Depletion Dynamics

In the case where the pyruvate is not replenished, the system we consider is (3), and we no longer impose $S_1\equiv 1$. However, in the no-replenishment case, the leading-order system is equivalent to the leading-order system for the continuous-replenishment case until $t= O (1/\epsilon )$. Thus, it is helpful to work in the long timescale $T= \epsilon t= O (1)$, over which the full system is given by (8b)–(8d), along with

36a $$\begin{aligned} \frac{\mathrm {d} S_1}{\mathrm {d} T}&= -\dfrac{ S_1}{\epsilon \left( S_1+ \bar{K}_1^M\right) + S_1S_2}, \end{aligned}$$

36b $$\begin{aligned} \frac{\mathrm {d} S_2}{\mathrm {d} T}&= \dfrac{ S_1}{\epsilon \left( S_1+ \bar{K}_1^M\right) + S_1S_2} - \dfrac{ \bar{k}_2S_2}{S_2+ \epsilon \bar{K}_{2}^M} + \dfrac{\bar{k}_{5}\hat{S}_4}{\epsilon \hat{S}_4+ \bar{K}_{5}^M} - \hat{A}S_2. \end{aligned}$$

### Slow Depletion of Pyruvate: $0< T< T_d$

Over the timescale $T= O (1)$ and until the pyruvate becomes of $O (\epsilon )$, the leading-order system for $S_2$, $\tilde{S}_4$, $\tilde{S}_3$, and $P$ is still given by (9), the leading-order system for the continuous-replenishment case. The leading-order equation for $S_1$ is obtained from (36a) to yield

37 $$\begin{aligned} \frac{\mathrm {d} S_1}{\mathrm {d} T} = -\dfrac{ 1}{ S_2}, \end{aligned}$$

which we can integrate to obtain the leading-order solution

38 $$\begin{aligned} S_1(T) = 1 -\int _0^{T} \! \dfrac{\mathrm {d}s}{ S_2(s)}, \end{aligned}$$

in terms of $S_2$. The solution (38) is valid until $S_1= O (\epsilon )$, at which point a new asymptotic balance occurs. This occurs when $T= T_d$ (equivalently $t= T_d/\epsilon$), where

39 $$\begin{aligned} S_1(T_d) = 0, \end{aligned}$$

with an $$ O (\epsilon )$$ correction (equivalently $$ O (1)$$ in terms of $$t$$). Hence, $$T_d$$ can be obtained by solving

40 $$\begin{aligned} \frac{\mathrm {d} S_2}{\mathrm {d} T} = \dfrac{1}{S_2} - \bar{k}_2+ \bar{v}_5\hat{S}_4(T) - \hat{A}S_2, \quad \text {with } S_2\sim \left( 2 T\right) ^{1/2} \text { as } T\rightarrow 0^{+}, \end{aligned}$$

forward in time, using (12), (14)–(15) for $\hat{S}_4$, and terminating the calculation when $S_1= 0$. Thus, this analysis is valid for $T< T_d$.

### Rapid Depletion of Pyruvate: $t- T_d/\epsilon = O (1)$

The next timescale is an interior layer (Holmes 2012), which occurs when $t- T_d/\epsilon = (T- T_d)/\epsilon = \bar{t} = O (1)$. Over this timescale, $S_1$ varies from being of $O (\epsilon )$ to exponentially small, while the remaining dependent variables are unchanged at leading order. Making the scaling $S_1= \epsilon Y$, the leading-order equation for the pyruvate concentration in this interior layer is given by

41 $$\begin{aligned} \frac{\mathrm {d} Y}{\mathrm {d} \bar{t}} = -\dfrac{ Y}{ \bar{K}_1^M+ Y S_2}, \end{aligned}$$

with appropriate matching conditions as $\bar{t} \rightarrow -\infty$. This depletion results in the exponential decay of *Y*, and hence $S_1$, and is a slightly modified version of the coupled pyruvate and acetyl-CoA depletion dynamics considered in Appendix B in Dalwadi et al. (2018). As these depletion dynamics are not required to calculate details of 3HP production, we do not consider the dynamics of $S_1$ any further here for brevity; the analysis will be similar to that in Dalwadi et al. (2018) and require first-order correction terms to match to an appropriate accuracy. Since $S_1$ is exponentially small as we move forward in time out of this interior layer, we henceforth treat it as zero in the remaining timescales.

### Slow Depletion of Acetyl-CoA: $T_d< T< T^*$

After the depletion of pyruvate, we remain in the timescale $T= t/\epsilon = O (1)$, but now with $T> T_d$. The system is thus given by (8b)–(8d), replacing (8a) with

42 $$\begin{aligned} \frac{\mathrm {d} S_2}{\mathrm {d} T} = - \dfrac{ \bar{k}_2S_2}{S_2+ \epsilon \bar{K}_{2}^M} + \dfrac{\bar{k}_{5}\hat{S}_4}{\epsilon \hat{S}_4+ \bar{K}_{5}^M} - \hat{A}S_2. \end{aligned}$$

As each non-pyruvate dependent variable remains unchanged over the previous interior layer, the correct matching conditions are obtained by imposing continuity of each dependent variable across $T= T_d$. The leading-order system is given by (9), replacing the first equation with

43 $$\begin{aligned} \frac{\mathrm {d} S_2}{\mathrm {d} T} = - \bar{k}_2+ \bar{v}_5\hat{S}_4- \hat{A}S_2. \end{aligned}$$

This system is solved by (11)–(12) and (15), along with

44 $$\begin{aligned} S_2(T) = - \dfrac{\bar{k}_2}{\hat{A}} + \left( S_2(T_d) + \dfrac{\bar{k}_2}{\hat{A}} \right) \hbox {e}^{-\hat{A}(T- T_d)} + \hbox {e}^{-\hat{A}(T- T_d)} \int _{T_d}^{T} \! \bar{v}_5\hat{S}_4(s) \hbox {e}^{\hat{A}(s - T_d)} \, \mathrm {d}s, \end{aligned}$$

and these solutions are valid until $S_2= O (\epsilon )$, at which point a new asymptotic balance will occur. This occurs when $T= T^*$ (equivalently $t= T^*/\epsilon$), where

45 $$\begin{aligned} 0&= S_2(T^*) = - \dfrac{\bar{k}_2}{\hat{A}} + \left( S_2(T_d) + \dfrac{\bar{k}_2}{\hat{A}} \right) \hbox {e}^{-\hat{A}(T^*- T_d)}\nonumber \\&\quad + \hbox {e}^{-\hat{A}(T^*- T_d)} \int _{T_d}^{T^*} \! \bar{v}_5\hat{S}_4(s) \hbox {e}^{\hat{A}(s - T_d)} \, \mathrm {d}s, \end{aligned}$$

with an $$ O (\epsilon )$$ correction (equivalently $$ O (1)$$ in terms of $$t$$). After this point, the remaining metabolites will all start to deplete, so $$P(T^*)$$ represents the maximal level of 3HP in the system with no replenishment of pyruvate. Physically, $$t = T^*/\epsilon $$ gives the asymptotic “optimal” time at which to terminate a batch run and harvest the 3HP that has been produced.

### The Remaining Depletion Dynamics

The remaining depletion dynamics are unimportant to the task of determining the maximal 3HP in the system, so we only state them briefly here for completion. The exact details of the transition of $$S_2$$ from $$ O (1)$$ to $$ O (\epsilon )$$ occur in another interior layer over the timescale $$t- T^*/\epsilon = (T- T^*)/\epsilon = O (1)$$, where $$\hat{S}_3$$ and $$\hat{S}_4$$ also vary but do not change in asymptotic order. Over this timescale, the levels of $$P$$ do not vary. In the forward-in-time far-field of this interior layer, the levels of each metabolite tend to a constant.

The final depletion dynamics occur over the timescale $T= t/\epsilon = O (1)$, but with $T> T^*$. Over this timescale, the metabolites decay exponentially, governed by an ODE for $P$ with the remaining metabolites coupled to the levels of $P$ in a quasi-steady manner.
